# Evaluation of a Stretching Forearm Sleeve for Lateral Epicondylitis: Repeated Measures Study

**DOI:** 10.2196/80400

**Published:** 2026-03-04

**Authors:** Adriana M Ríos Rincón, Christine Guptill, Ann Tran, Rija Kamran, Salamah Alshammari, Antonio Miguel Cruz

**Affiliations:** 1 Department of Occupational Therapy Faculty of Rehabilitation Medicine University of Alberta Edmonton, AB Canada; 2 Department of Occupational Therapy University of Ottawa Ottawa, ON Canada; 3 Occupational Therapy Program College of Applied Medical Sciences King Saud bin Abdulaziz University for Health Sciences Alahsa, Eastern Region Saudi Arabia; 4 Glenrose Rehabilitation Research, Innovation & Technology Glenrose Rehabilitation Hospital Edmonton, AB Canada

**Keywords:** lateral epicondylitis, musculoskeletal, orthopedic device, rehabilitation, tennis elbow, upper extremity

## Abstract

**Background:**

Lateral epicondylitis (LE) is a condition that impairs daily activities due to pain exacerbated by wrist and hand movements. The ArmLock sleeve is a novel, nonsurgical intervention to stretch the wrist extensor muscles by maintaining the elbow in extension, forearm in pronation, and wrist and fingers in flexion.

**Objective:**

This study aimed to assess the effectiveness of sustained tension provided by the ArmLock sleeve on pain and functional outcomes in adults with LE. This novel device supports the forearm by aligning the elbow in extension, the forearm in pronation, and the wrist in flexion, while keeping the metacarpophalangeal and proximal interphalangeal joints of fingers II-V flexed.

**Methods:**

A within-subjects repeated-measures design was used to assess outcomes at baseline, week 6, and week 12. Nineteen participants wore the device at home for 30 minutes daily for 12 weeks. Outcome measures were administered by research assistants and included pain intensity, pain-free grip strength, pressure pain threshold, pain during resisted wrist extension, and composite extensibility of wrist and finger extensors. Repeated-measures 1-way ANOVA and Friedman tests were conducted (α=.05), followed by post hoc comparisons (α=.017, Bonferroni correction).

**Results:**

Significant improvements were observed in 6 of 7 (85.7%) outcome variables, including increased muscle extensibility, enhanced grip strength, and reduced pain intensity. Participants reported decreased pain and functional disability in a self-reported questionnaire.

**Conclusions:**

Wearing the device daily for 12 weeks led to significant improvements in extensibility, grip strength, and pain reduction. Participants also reported decreased pain and disability. These results suggest that the ArmLock sleeve may support symptom relief and functional gains in individuals with LE. Larger, controlled studies are needed to confirm its effectiveness.

**Trial Registration:**

ISRCTN Registry ISRCTN13309889; https://www.isrctn.com/ISRCTN13309889

## Introduction

Lateral epicondylitis (LE), known as tennis elbow, is a frequent cause of lateral elbow pain that significantly impacts the performance of work, self-care, and leisure activities, as the pain intensifies with wrist and hand movements [1]. Aggravating movements often include resisted wrist extension, middle finger extension, and forearm supination with a fully extended elbow [2]. Prevalence studies indicate that LE affects approximately 1.3% of the general population [1], with higher rates observed in working populations (2%-14.5%) [3] and among tennis players (14%-41%) [4]. The condition is predominantly seen in the dominant upper extremity of individuals aged 30-64 years, with peak incidence occurring between ages 45 and 54. LE is strongly associated with repetitive and forceful tasks [1]. As an increasing percentage of the aging population remains physically active, the incidence of LE is expected to rise significantly in the coming years [5]. Given the high prevalence of LE, its socioeconomic impact can be substantial. For example, in the United States, nontraumatic epicondylitis accounts for an annual workers’ compensation claim incidence rate of 10.6 per 10,000 full-time equivalent employees [6].

The etiology of LE remains a subject of ongoing debate within the medical community [7]. The most widely accepted causative factor is repetitive microtrauma, resulting from overuse of the wrist extensor or supinator muscles. Such overuse commonly arises from prolonged heavy workloads, intensive training in racquet sports, or improper technique during physical activities [7,8]. While LE has historically been described as a tendinosis based on histopathological findings [7], contemporary models conceptualize the condition as a degenerative tendinopathy characterized by maladaptive responses to mechanical loading, altered mechanobiology, and pain mechanisms that extend beyond local tissue pathology [9]. This distinction has important therapeutic implications, supporting interventions that emphasize load modulation, sustained low-load mechanical stimuli, and functional restoration rather than anti-inflammatory approaches alone. As a degenerative tendinopathy, LE is characterized by maladaptive responses to mechanical loading, which may include excessive or uneven tensile forces acting on the tendon and contributing to microtrauma and degenerative changes [9,10]. In degenerative tendons, the typically organized structure of collagen architecture is disrupted, with fibroblasts and atypical vascular tissue creating a tangled, disorganized matrix with reduced tensile capacity [10]. LE involves chronic tendon degeneration at the common origin of the forearm extensor muscles. These muscles include the extensor carpi radialis brevis (ECRB), extensor digitorum, extensor digiti minimi, and extensor carpi ulnaris, with the ECRB tendon most frequently affected at the lateral epicondyle of the humerus [9,10].

While many individuals with LE experience symptom resolution over time, including spontaneous improvement without formal treatment, most also demonstrate a positive response to conservative management. However, a clinically meaningful subset of patients does not achieve symptom resolution. Approximately 5%-10% of individuals develop persistent symptoms, with pain extending beyond several weeks, exceeding what would be expected from spontaneous recovery alone and suggesting progression toward a chronic or recalcitrant presentation. Patients whose symptoms persist despite appropriate rehabilitation interventions, particularly beyond 3-6 months, are commonly classified as having recalcitrant LE. In this subgroup, continued passive observation is unlikely to yield meaningful improvement, underscoring the need for targeted interventions aimed at modifying tendon loading and mechanobiological processes [9].

A wide range of rehabilitation and nonsurgical interventions has been described for LE, including corticosteroid injections, iontophoresis, botulinum toxin A injections, prolotherapy, platelet-rich plasma (PRP) or autologous blood injections, as well as physical and occupational therapy approaches such as bracing and splinting, stretching and strengthening programs, kinesiotaping, extracorporeal shockwave therapy, and laser therapy. Among these, PRP has been identified as a conservative treatment with higher levels of supporting evidence [11,12]. However, current research does not clearly demonstrate that PRP is superior to other nonsurgical approaches [11,12]. Surgical intervention is typically reserved for approximately 3% of patients who fail to respond to nonsurgical treatments after 1 year from symptom onset [13].

Although the evidence remains somewhat ambiguous, rehabilitation interventions are generally recommended as first-line treatment over medications, corticosteroid injections, or surgery [14]. Braces and splints represent a rehabilitation intervention for LE, particularly for individuals with limited access to other rehabilitation services. These devices aim to reduce mechanical load on the extensor tendons by providing external support. Commonly used orthoses include forearm straps, which decrease force transmission at the common extensor origin, and wrist extension splints, which offload the extensor musculature by immobilizing the wrist. However, evidence regarding their effectiveness in alleviating LE symptoms remains inconclusive [1]. Therapeutic exercise, including stretching, is supported for short-term improvements in pain and function; however, there is no clear consensus on an evidence-based multimodal rehabilitation program for LE, nor on optimal exercise dosage parameters, including intensity, duration, frequency, and progression [14].

Stretching exercises targeting the wrist extensor musculature are commonly incorporated as part of multimodal conservative management strategies for LE [14-16]. A frequently described technique involves wrist flexion with ulnar deviation, combined with elbow extension and forearm pronation, with or without concurrent finger flexion [14,17]. Although the direct effects of static stretching on tendon structure remain incompletely understood, mechanical loading is recognized as an important stimulus for tendon cell activity. Experimental and clinical literature suggests that the mechanical load generated by this upper limb position may support tendon healing in LE by encouraging fibroblasts to align new collagen fibers in an orderly manner, improving the tendon’s structural integrity [18]. Accordingly, the mechanical load generated during wrist extensor stretching is hypothesized to contribute to these cellular responses, although the magnitude and durability of such effects in degenerative tendinopathy remain variable and require further investigation.

A promising rehabilitation intervention for LE is the ArmLock sleeve, which applies principles used in therapeutic stretching. The device positions the elbow in extension, the forearm in pronation, and the wrist in flexion, while maintaining flexion of the metacarpophalangeal and proximal interphalangeal joints of digits II-V. This configuration reproduces a frequently prescribed stretching posture used in multimodal rehabilitation programs and is intended to apply sustained mechanical loading to the musculotendinous structures implicated in LE. Specifically, this alignment targets the ECRB, extensor digitorum, extensor digiti minimi, extensor carpi ulnaris, and the superficial head of the supinator, which attach near the lateral epicondyle. By providing prolonged, externally supported positioning, the ArmLock sleeve may offer a practical means of delivering consistent mechanical stimulus as part of a rehabilitation approach, particularly when adherence to repeated active stretching is challenging. However, the effectiveness of the ArmLock sleeve in reducing LE symptoms has not yet been determined. This study aimed to assess the effectiveness of sustained tension provided by the ArmLock sleeve on pain and functional outcomes in adults with LE.

## Methods

### Study Research Design

A within-subjects repeated-measures design was used to address the research objective. Outcomes related to pain and function were assessed at 3 time points: baseline (week 0), midintervention (week 6), and postintervention (week 12). The use of a within-subjects repeated-measures design to evaluate the effects of the ArmLock sleeve on pain and function in adults with LE aligns with contemporary frameworks for generating evidence in rehabilitation technologies and reflects the technology’s level of maturity. Specifically, this study was guided by the Framework for Accelerated and Systematic Technology-based Intervention Development and Evaluation Research (FASTER), which cautions against the routine application of pharmaceutical-style randomized controlled trials (RCTs) to early-stage, rapidly evolving technology-based interventions in disability and rehabilitation [19]. Increasingly, such expectations have been questioned due to their high cost and logistical burden, the risk that extended trial timelines render technologies obsolete, and the limited external validity of tightly controlled experimental conditions. FASTER therefore recommends quasi-experimental designs as rigorous and appropriate approaches for phase 2 (progressive usability and feasibility evaluation). Within-subject repeated-measures designs, in particular, are well suited to this phase because they allow participants to serve as their own controls while also enabling group analysis, thereby facilitating the examination of individual change trajectories and effect signals. The effect sizes and response patterns observed in this study provide critical parameters for subsequent sample size estimation, refinement of research protocol, and hypothesis development in future controlled trials. By situating this study within an iterative, technology-specific evidence-generation pathway, consistent with the FASTER framework, our findings contribute to generating foundational evidence, including effect size estimates, necessary to design future RCTs.

### Materials

**Figure 1:**
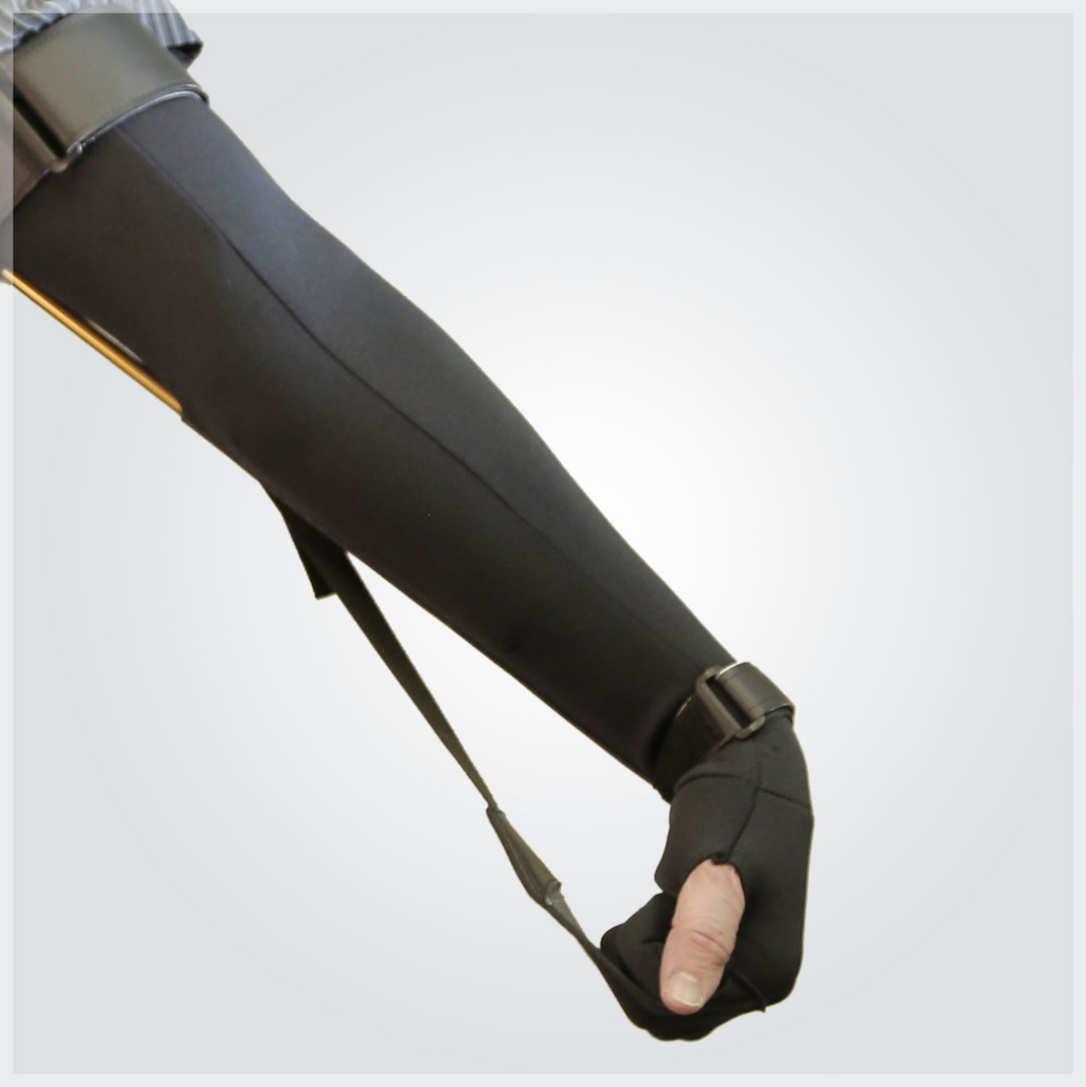
The ArmLock sleeve.

As illustrated in Figure 1, the ArmLock sleeve is a soft upper-limb orthosis designed to provide sustained positioning and postural support of the elbow, forearm, wrist, and hand. The orthosis incorporates an adjustable strapping system within a semistructured textile sleeve to maintain the elbow in extension, the forearm in pronation, and the wrist in flexion, while positioning the metacarpophalangeal and proximal interphalangeal joints of digits II-V in flexion. The orthosis is fabricated from a breathable, elastic textile composite (74% polyester, 20% rayon, and 6% elastic). Its design allows adjustable traction to increase or decrease wrist and finger flexion to the degree required to achieve the intended positioning. The ArmLock sleeve is classified as a class I medical device, is licensed by Health Canada (Medical Device Establishment Licence [MDEL] No. 7429), and is commercially available.

### Participants

Participants were eligible for inclusion if they were 18 years or older, able to communicate in English, and had a self-reported diagnosis of LE accompanied by the following symptoms: (1) pain on the lateral side of the elbow elicited by resisted wrist extension, (2) tenderness at the lateral epicondyle, (3) measurable loss in composite muscle flexibility (extensibility), and (4) symptoms persisting for at least 12 weeks.

Exclusion criteria were applied to minimize confounding factors and ensure the safety and appropriateness of participants for this study. Potential participants were excluded if they (1) had a steroid injection for LE within the past 3 months (steroid injections are a treatment for LE and could confound the study results) and (2) had undergone previous surgery for LE (approximately 10% of postsurgical cases remain symptomatic, suggesting severe LE or underlying conditions unrelated to this study) [9].

Exclusion criteria for symptoms likely due to other medical conditions. These conditions could mimic or contribute to LE-like symptoms or reflect different underlying pathologies, making them unsuitable for this study. Such exclusions included those who (1) had been diagnosed with elbow, wrist, or finger arthritis; (2) had sensory and/or motor changes distal to the elbow, such as carpal tunnel syndrome or elbow joint instability; (3) experienced pain associated with radiculopathy, cervical nerve compression, or thoracic outlet syndrome; and (4) practiced high-velocity racquet sports, such as tennis or badminton.

Exclusion criteria for conditions preventing safe or appropriate use of the ArmLock sleeve included those who (1) exhibited a difference of less than 15 degrees in the range of motion (ROM) of wrist flexion with fingers extended compared to fingers flexed as such cases indicate limited capacity for further mechanical stretching using the ArmLock sleeve; (2) experienced pain during ligament stress tests at the elbow, which may indicate ligament instability, precluding safe use of the device; and (3) had a wound or scarring in the area where the ArmLock sleeve would be applied as these conditions could prevent proper application and pose risks to healing.

### Settings

All assessments were performed at Lab 1-45 Corbett Hall, University of Alberta. Participants took the Armlock sleeve home.

### Sample Size

We aimed to recruit 31 participants with a power of 0.8, an alpha of .05, and a medium effect size (f=0.30) [20]. The sample size calculation was based on the Patient-Rated Tennis Elbow Evaluation (PRTEE). In previous studies, sample sizes between 28 and 33 participants were enough to detect a change in the PRTEE (*P*=.002) [21].

### Recruitment

The sampling strategy used was nonprobabilistic. Study details were disseminated through various channels, including social media platforms, online classified advertisement services, regional newspapers, and flyers. Flyers were placed in multiple locations, such as bulletin boards at postsecondary institutions, orthopedic supply stores, and medical supply retail stores. Additionally, the research team reached out to local private clinics and professionals using email lists available on professional association websites in Alberta. Potential participants who expressed interest in the study contacted the research team for more information.

Once participants initiated contact, a member of the research team provided a detailed explanation of the study over the phone and conducted screening questions using a standardized telephone script to confirm eligibility. If an individual met the inclusion criteria and expressed interest in participating, the research assistant emailed the information letter for further review and invited them to an in-person session for upper extremity screening. During this session, researchers explained that the purpose of the screening was to confirm eligibility through a physical evaluation. Participants provided signed consent before proceeding with the assessment. The upper extremity screening included (1) isometric resisted muscle testing, (2) sensory examination (light touch), and (3) ROM assessment of the wrist and fingers.

Any asymmetry between sides or a ROM limitation that prevented the participant from tolerating the position required to wear the ArmLock sleeve resulted in exclusion from the study.

Additionally, the following clinical tests were performed:

1. Elbow Varus Instability Stress Test (to rule out elbow joint instability) [22]
2. Spurling Test (to assess cervical nerve compression) [23]
3. Roos Test (to assess thoracic outlet compression) [24]
4. Upper Limb Reflex Testing (to rule out radiculopathy) [25]

If a potential participant tested positive for any of these assessments, they were deemed ineligible for the study, and the research team informed them that the ArmLock sleeve would not be beneficial for their condition. The research team thanked the participants for their time and provided parking compensation for their participation.

### Variables

#### Dependent Variables (Outcome Variables)

A combination of objective and subjective (self-reported) outcome variables was used to assess the effectiveness of the ArmLock sleeve intervention. The following outcome variables were measured:

#### Composite Wrist and Finger Extensor Muscles

The composite extensibility of the wrist and finger extensor muscles is the muscle tightness of the wrist and finger extensors (objective outcome variable). For this measure, we followed an adaptation of the Mill Test for LE. Participants were positioned with a closed hand, the wrist in dorsiflexion, and the elbow in extension. The examiner then applied wrist flexion, instructing the participant to resist the movement. The test was considered positive and discontinued when the participant reported pain at the lateral epicondyle [26]. No overpressure was applied, and wrist flexion was measured using a 12-inch clear plastic goniometer (Baseline 360°). The value was recorded in degrees.

#### Pain-Free Grip Strength

Pain-free grip strength (PFG) is the maximal grip strength at which pain appears (objective outcome variable). This is a common measure of pain in LE. In this study, we followed the protocol published in previous studies of tennis elbow [4,27]. First, to minimize the risk of exacerbating injuries during PFG assessment, participants were first introduced to the concept of PFG. Then, participants were asked to stand with the elbow in a complete extension and the shoulder and radioulnar joints in neutral rotation. They were then instructed to slowly squeeze the dynamometer to their maximum strength using the unaffected arm, allowing them to become familiar with the device. Following this, participants repeated the process with the affected arm, maintaining the same gripping rate as the unaffected side but stopping immediately upon experiencing any discomfort. We asked the participants to perform 3 trials with 1-minute rest intervals, as done in previous tennis elbow studies [4,27]. PFG was measured using the Sammons Jamar Plus+ digital hand dynamometer (200-lb capacity; Performance Health, Sammons Preston). The value was recorded in pounds (lb).

#### Pressure Pain Threshold

The pressure pain threshold (PPT; objective outcome variable) was defined as the minimum amount of pressure required to elicit pain. Participants were seated facing a table with their forearms resting on its surface. The researcher identified the muscle belly of the wrist extensor group by asking the participant to repeatedly extend their middle finger. Testing began on the unaffected side and was then repeated on the affected side, alternating between sides for a total of 3 measurements. A 20-second interval was provided between each measurement. The PPT was measured using the Commander Echo digital algometer with a 1-cm² rubber probe tip (JTECH Medical), applied to the most tender point over the lateral epicondyle, following the method described in a study by Cho et al [4]. The value was recorded in pounds (lb).

#### Pain During Resisted Wrist Extension

Pain during resisted wrist extension (2 lb) test (subjective outcome variable) is a common symptom of LE. For this assessment (pain with lifting a 2-lb weight), participants were instructed to hold a 2-lb weight while standing with their arm relaxed. They were then asked to slowly lift the weight by flexing the elbow from 0 to 120 degrees and subsequently return to the starting position, following the method described by Cho et al [4]. Pain experienced during the movement was rated by the participant using a Numerical Rating Scale (NRS) from 0 to 10, with 0 = “no pain” and 10 = “worst possible pain.”

#### Pain and Functional Disability

Pain in the affected arm and functional disability were assessed using the PRTEE (subjective [self-reported] outcome variable). The PRTEE is a 15-item questionnaire that evaluates 2 key components: pain (5 items) and functional disability (10 items). The functional disability section is further divided into 2 parts: specific daily activities (eg, turning a doorknob or key, pulling up pants; 6 items) and usual activities (eg, personal care, household tasks, work, and recreational activities; 4 items). The PRTEE is a widely used, validated assessment tool with excellent psychometric properties [28,29]. The questionnaire provides subscale scores for pain and function, as well as a total score, all ranging from 0 to 100. The total score is the sum of the pain and functional disability subscales (maximum score 100). Higher scores indicate greater symptom severity. In this study, we used the pain subscale, the functional disability subscale, and the total score as outcome variables.

#### Independent Variable

The independent variable in this study was the use of the ArmLock sleeve. Participants were instructed to wear the device for 30 minutes daily for a 12-week period as an intervention to treat LE. The prescribed duration of ArmLock sleeve use was informed by clinical guidance for stretching protocols in lateral elbow tendinopathy and by parameters reported in prior RCTs. Clinical guidelines commonly describe stretching protocols involving multiple repetitions of 30-45 second holds, typically performed twice daily [30]. Across randomized trials, stretching parameters vary substantially, with hold durations ranging from 6 to 45 seconds, delivered over multiple sets (eg, 1-3), multiple repetitions (eg, 6-15), and performed 2 to 3 times per day [15].

Acknowledging this variability, the decision to prescribe 30 minutes of daily ArmLock sleeve use represented an informed estimation of these parameters, corresponding to an approximate cumulative stretch exposure of ~10-33 minutes when considering total time under stretch across repetitions and sessions reported in the literature. For this study evaluating the effectiveness of a novel technology, we intentionally selected a duration toward the upper end of reported parameter ranges to ensure sufficient stretch exposure while also examining participant tolerance to a sustained, cumulative stretching dose that approximates or exceeds conventional protocols. This approach allowed us to balance theoretical therapeutic dosing with feasibility, safety, and tolerability considerations in our evaluation.

#### Baseline Participant Information

Participants completed a demographic questionnaire that collected information on age, gender, occupation, side affected, current treatments (eg, hand therapy), and symptom duration.

### Procedures

Participants who met the inclusion criteria and provided written consent were invited to attend an initial meeting. During this first meeting, participants completed the demographic questionnaire. A research assistant administered the measures of each outcome variable. We started with the ones that were not painful at all and finished with those that were potentially the most painful for participants. The order of the tests was as follows: PRTEE, composite extensibility, PFG, the pain with lifting a 2-lb weight, and PPT. After these assessments, the research assistant provided each participant with an ArmLock sleeve and instructed them on how to properly wear it. Participants wore the device for 15 minutes to check for any signs of skin irritation (eg, red marks or pressure points) or discomfort. The device was adjusted as needed. Participants were instructed to wear the ArmLock sleeve for 30 minutes daily for a 12-week period, at a time of their choosing. Device use was documented using a Daily Log Sheet provided by the research team. For each session, participants recorded the time the sleeve was donned and doffed, the total duration of wear, and any relevant comments (eg, pain, discomfort, or events that prevented adherence to the recommended 30-minute wear time). Participants were asked to return the completed Daily Log Sheet at their subsequent study visit. The research team used the logged information to monitor adherence and document participant-reported experiences with device use.

Participants attended 2 additional sessions: one at week 6 and a final session at week 12. During these visits, the researcher readministered all outcome measures. At the week 12 session, a brief exit interview was conducted to gather participants’ feedback on the use and acceptance of the ArmLock sleeve. Additionally, follow-up phone calls were made during weeks 3 and 9 to monitor participants’ use of the device and to remind participants to track use in the Daily Log Sheet.

### Statistical Analysis

We described the demographics by reporting the frequency and proportion for categorical variables and the mean (SD) for continuous variables. To determine the normality of the data, histogram analysis and the Shapiro-Wilk and Kolmogorov-Smirnov tests were conducted. Repeated-measures one-way ANOVA and Friedman tests were conducted to evaluate differences among the measurement time points (week 0, week 6, and week 12) for normally distributed and non-normally distributed data, respectively. Post hoc comparisons between the measurement time points were evaluated using paired *t* tests and Wilcoxon signed-rank for normally distributed and non-normally distributed data, respectively. Bonferroni post hoc correction was used to account for the increased risk of type I errors associated with multiple comparisons. A *P* value <0.05 was considered to indicate statistical significance for ANOVA and Friedman tests. The Bonferroni correction level of statistical significance was corrected at a *P* value <.02 (ie, 0.05/3 tests per outcome variable). All the statistical analyses and visual presentations were performed using IBM SPSS (version 29.0.2.0).

### Ethical Considerations

The University of Alberta Research Ethics Board reviewed and approved this study (Pro00095330). Potential participants contacted the research team to express interest, and written consent was obtained before participation. All personal data were deidentified prior to data analysis.

Participants received parking compensation for the time spent in each in-person session, including the Upper Extremity Screening for eligibility determination and the assessment sessions at weeks 0, 6, and 12. Additionally, participants received the ArmLock sleeve at no cost and were allowed to keep it after the study was completed.

## Results

### Participant Flow

**Figure 2:**
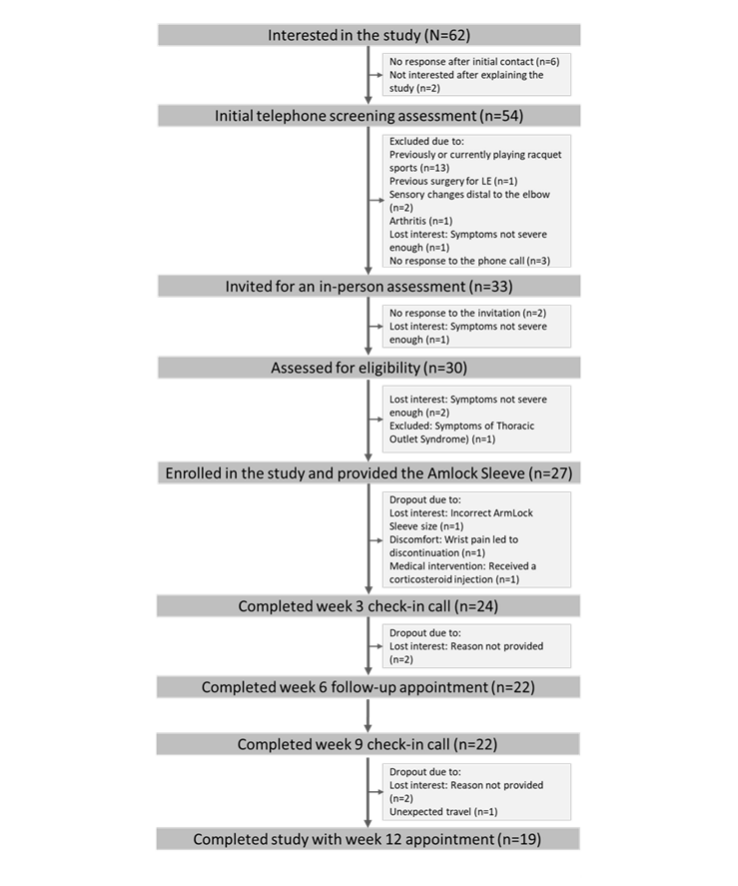
Study flowchart. LE: lateral epicondylitis.

Figure 2 illustrates the progression of participants throughout the study, between March 23, 2020, and April 30, 2023. A total of 62 individuals from the community contacted the researchers to express interest in participating. Of these, 54 individuals took part in an initial telephone screening assessment, with 21 excluded at this stage. Subsequently, 30 participants proceeded to an in-person eligibility assessment, where 2 declined to participate and 1 was excluded. Ultimately, 27 participants enrolled in the study, completed baseline assessments and received the ArmLock sleeve. All enrolled participants were scheduled to have follow-up assessments conducted at week 6 and week 12. Throughout the study, 8 participants dropped out for various reasons, as detailed in Figure 2. In total, 19 participants completed the study, and their data were included in the final analysis. Based on this participant’s flow, the study retention was 70% (19/27). Compliance with the intervention was measured based on the number of days the device was used and the duration of daily use. On average, participants used the device for 62 days, representing 74% of the recommended 84 days. The average daily usage time was 30 (SD 2.62) minutes.

### Demographics of Participants

Table 1 presents the demographic characteristics of participants. The majority of participants were middle-aged adults (mean age 44.11, SD 10.57 years), male, and employed. The most commonly reported treatment strategies used in the past to manage tennis elbow included performing ROM exercises and using a brace or elbow strap.

At the time of data collection, nearly half of the participants reported engaging in ROM exercises, approximately one-quarter were taking nonsteroidal anti-inflammatory drugs such as ibuprofen, aspirin, or naproxen, and slightly more than one-quarter reported no nonsurgical concomitant treatments. Additionally, slightly more than half of the participants reported that LE symptoms lasted for 1 hour or more.

**Table 1:** Demographics of participants (N=19).

Variable			Participants, n (%)
**Sex**			
	Male	13 (68.4)	
	Female	6 (31.6)	
**Employment status**			
	Employed	9 (47.4)	
	Self-employed	2 (10.5)	
	Out of work and looking	3 (15.8)	
	Out of work but not looking	1 (5.3)	
	Student	1 (5.3)	
	Retired	2 (10.5)	
	Unable to work	1 (5.2)	
**Previous treatment**			
	Range of motion exercises	13 (68.4)	
	Brace or elbow strap	13 (68.4)	
	Nonsteroidal anti-inflammatory drugs	10 (52.6)	
	Ice	10 (52.6)	
	Physiotherapy or massage therapy	9 (47.4)	
	Steroid injections	1 (5.3)	
**Present treatment**			
	Range of motion exercises	9 (47.4)	
	Nonsteroidal anti-inflammatory drugs	5 (26.3)	
	Ice	3 (15.8)	
	Physiotherapy or massage therapy	3 (15.8)	
	Brace or elbow strap	3 (15.8)	
	Steroid injections	0 (0)	
**Duration of symptoms**			
	One hour or more	11 (57.9)	
	Less than 1 hour	8 (42.1)	

### Effectiveness of the ArmLock Sleeve on Reducing LE Symptoms

Table 2 presents the descriptive statistics and results of the Friedman tests for the outcome variables across measurement time points. The analysis revealed statistically significant changes in 85.7% (6/7) of the outcome variables, suggesting that the ArmLock sleeve was effective in improving several key measures. Specifically, the intervention resulted in increased composite extensibility of the wrist and finger extensor muscles, enhanced PFG, and reduced pain intensity when lifting a 2-lb weight. Additionally, participants reported decreases in self-reported pain and functional disability, as measured by the PRTEE, with significant improvements observed in the pain subscale, the functional disability subscale, and the total score.

**Table 2:** Descriptive statistics and Friedman test for the outcome variables across measurement time points (week 0, 6, and 12; N=19)^a^.

Outcome variable	Descriptive statistics									Friedman test results				
	Week 0			Week 6			Week 12			Chi-square (*df*)		*P* value		W^b^
	Mean (SD)	Median (IQR)	Mean (SD)		Median (IQR)	Mean (SD)		Median (IQR)						
Ext-AA^c^ (degrees)	45.0 (8.6)	45.0 (40.0-54.0)	48.6 (5.1)		49.0 (46.0-53.0)	48.0 (8.8)		51.0 (43.0-55.0)	6.08 (2)		*.048*		0.160	
PFG-AA^d^ (lb)	48.9 (29.9)	43.1 (20.9-76.4)	64.2 (25.5)		59.0 (42.7-88.3)	73.2 (22.9)		76.0 (65.6-88.9)	27.26 (2)		<*.001*		0.717	
PPT-AA^e^ (lb)	4.1 (1.1)	3.9 (3.2-5.1)	4.3 (1.1)		4.9 (3.4-4.6)	4.7 (1.7)		4.2 (3.5-6.0)	4.89 (2)		.09		0.129	
PWL 2lb-AA^f^	3.16 (2.2)	3.00 (1.0-4.0)	1.7 (2.5)		1.0 (0.0-3.0)	0.84 (1.8)		0.0 (0.0.-1.0)	24.00 (2)		<*.001*		0.632	
PAINS_SUB^g^	22.0 (8.2)	20.00 (16.0-30.0)	12.5 (9.0)		11.00 (5.0-17.0)	7.9 (9.5)		5.0 (2.0-8.0)	27.26 (2)		<*.001*		0.717	
FUNC_SUB^h^	19.6 (10.8)	24.0 (8.5-30.5)	9.7 (9.9)		5.0 (3.0-15.0)	6.0 (9.5)		3.0 (1.0-5.5)	29.95 (2)		<*.001*		0.788	
TOTAL_W^i^	41.6 (17.7)	46.0 (27.0-59.0)	22.2 (18.6)		9.0 (9.0-32.5)	14.0 (18.7)		8.0 (4.0-13.0)	28.74 (2)		<*.001*		0.756	

^a^Italicized *P* values indicate statistical significance at *P*<.05.

^b^W: Kendall’s coefficient of concordance (considered a measure of effect size in the context of the Friedman Test). ^c^Ext-AA: composite extensibility of wrist and finger extensor muscles–affected arm.

^d^PFG-AA: pain-free grip strength–affected arm.

^e^PPT-AA: pressure pain threshold–affected arm.

^f^PWL 2lb-AA: pain with lifting a 2-lb weight–affected arm.

^g^PAINS_SUB: Patient-Rated Tennis Elbow Evaluation pain subscale.

^h^FUNC_SUB: Patient-Rated Tennis Elbow Evaluation functional disability subscale.

^i^TOTAL_W: Patient-Rated Tennis Elbow Evaluation total scale.

**Figure 3:**
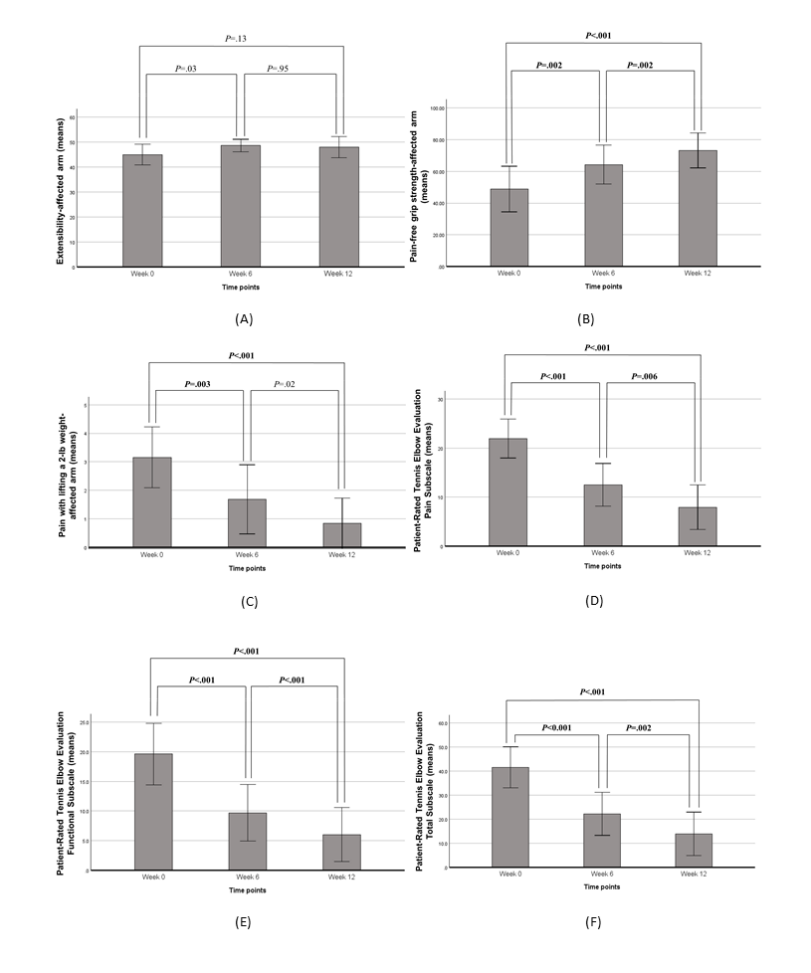
Post hoc comparisons between time points. The *P* values in the figure are obtained from the Wilcoxon signed-rank tests.

Figures 3A-3F illustrates the mean values of each outcome variable for the affected arm across the 3 time points (week 0, week 6, and week 12), along with post hoc comparisons between time points using Bonferroni correction. The pain pressure threshold was excluded from the post hoc comparisons, as the Friedman test did not indicate statistically significant changes for this variable. Error bars represent the SEM at each time point.

In Figure 3, Bonferroni correction: α _adjusted_ = .017. The α_adjusted_ was calculated as follows: α_adjusted_ =/*m*, where *m* is the number of tests per outcome variable (ie, *m*=3) and =.05. All the *P* values in bold are statistically significant using the new α_adjusted_.

For composite extensibility of the wrist and finger extensor muscles, the results suggest an initial improvement between week 0 and week 6, followed by stabilization from week 6 to week 12 (Figure 3A). However, despite the observed improvement at week 6, the changes from week 0 to week 6 and week 0 to week 12 were not statistically significant.

PFG showed a progressive improvement over time (Figure 3B), with statistically significant changes at each stage. These findings reinforce the effectiveness of the ArmLock sleeve in enabling participants to achieve a stronger, pain-free grip. The decreasing *P* values indicate that the cumulative improvement from week 0 to week 12 was greater than the improvement observed over shorter intervals.

Pain during resisted wrist extension, measured by the pain with lifting a 2-lb weight test, significantly decreased at each consecutive time point, suggesting a consistent reduction in pain throughout the 12-week period (Figure 3C). The lowest *P* value (*P*<.001) for the comparison between week 0 and week 12 confirms that the overall reduction in pain across the study period was statistically significant.

The subjective assessment of tennis elbow aligned with the objective measures. As shown in Figure 3E and F, both the pain subscale and functional disability subscale of the PRTEE decreased over time, indicating that the ArmLock sleeve had a positive impact on pain reduction and functional limitations in daily activities. Consequently, the total PRTEE score also declined over time, with all observed changes being statistically significant.

Because participants were not restricted from engaging in nonsurgical concomitant care, potential confounding effects were further examined analytically by including concomitant treatment status (yes or no) as a between-subjects factor in a repeated-measures ANOVA. This analysis evaluated whether changes in pain and functional outcomes over the intervention period differed between participants who did (n=14) and did not (n=5) receive concomitant treatments. The results indicated that pain pressure threshold was the only outcome significantly influenced by concomitant treatment status (β=17.073; *P*=0.03; partial η²=0.250). Notably, pain pressure threshold was also the only outcome that did not demonstrate a significant change over time in response to the ArmLock sleeve intervention. Taken together, these findings suggest that concomitant treatments did not meaningfully confound the observed intervention-related improvements in the remaining outcomes.

## Discussion

### Principal Findings

This study aimed to evaluate the effects of sustained tension provided by the ArmLock sleeve on biomechanical and functional outcomes in adults diagnosed with LE. The results of this within-subjects repeated-measures design study provide evidence of the potential clinical benefits of the ArmLock sleeve in reducing pain and improving function. Despite the small sample size, both subjective and objective measures of pain and functional disability outcomes demonstrated statistically significant reductions, with large effect sizes (W≥0.5). Improvements were observed in composite extensibility of the wrist and finger extensor muscles, PFG, pain with lifting a 2-lb weight, and the pain and functional disability subscales of the PRTEE. These findings suggest that the sustained muscle stretching provided by the ArmLock sleeve contributed to a substantial reduction in symptoms and enhanced functional performance in individuals with LE.

PFG refers to the maximum force an individual can exert during a gripping action without experiencing pain. It is a widely recommended measure for assessing function and treatment progress in individuals with LE [31]. PFG has also been found to be highly correlated with self-reported pain and has demonstrated high accuracy in detecting clinically important changes over time in individuals with LE [32]. Therefore, the significant increase in PFG observed in our study for the 12-week period is supported by a valid and reliable measure capable of detecting meaningful changes in patients with LE.

Hill et al [33] identified a minimal detectable change for PFG in individuals with LE of 9.2 kg force, which is approximately 20.28 lb. In our study, the observed increase in PFG for 12 weeks was 24.3 lb, exceeding the minimal detectable change threshold of 20.28 lb. This suggests that the measured improvement is clinically meaningful and unlikely to be attributed to measurement error.

The results from objective measures align with subjective assessments, reinforcing the intervention’s effectiveness. Findings from the PRTEE indicate that participants experienced notable improvements, including reduced pain and discomfort and enhanced arm functionality in daily activities. During the exit interview, participants reported resuming various activities due to the ArmLock sleeve intervention, such as playing hockey and golf, opening jars, typing, picking up a cup of coffee, putting on pants, opening doors, snow shoveling, paddling a canoe, climbing, and carrying heavy bags or groceries. However, 2 (11%) participants reported no perceived improvements with device use. Variations in the device’s effectiveness may be influenced by lifestyle factors such as smoking and alcohol consumption, which have been associated with nonsurgical treatment failure [34].

The majority of participants found the ArmLock sleeve easy to put on and remove, indicating good usability. However, some participants faced challenges in adjusting the optimal tightness and correctly using the straps. Among those who completed the study, 3 (16%) participants reported numbness, tingling, or soreness while wearing the device. One participant noted that these symptoms resolved after loosening the wrist strap. Additionally, 1 participant withdrew from the study due to wrist pain. These findings highlight the need for future research to explore potential adverse effects associated with wearing the device and to optimize its design and usage guidelines.

The high retention rate and compliance with the intervention indicate that participants accepted the ArmLock device and tolerated the recommended daily wearing time of 30 minutes. Participants provided feedback on how they adjusted their schedules to accommodate device use, and many appreciated the ability to wear it privately at home. Several participants expressed satisfaction with the outcomes of using the device daily. However, not all were able to do so consistently due to various challenges, including time constraints, forgetfulness, evening fatigue, or a diminished perception of the stretching effect after several weeks of use. These insights highlight the importance of considering individual adherence factors in future implementations of the device.

Finally, the ArmLock sleeve controlled and gentle sustained stretch may promote collagen fiber alignment and increase blood flow, thereby facilitating tendon healing. This mechanism is consistent with the observed reductions in pain and improvements in function demonstrated in this study.

### Study Limitations

This study has some limitations. First, the small sample size was influenced by recruitment challenges, particularly in a community setting where potential participants were not referred by health care professionals. Additionally, 15 individuals who initially expressed interest did not respond to follow-up calls or invitations. Future studies should consider recruiting from hospitals and clinics to improve participant retention and sample size. The COVID-19 pandemic further impacted recruitment, delaying the study for 1 year due to restrictions on in-person screening. Second, the lack of a control group limits the ability to attribute improvements solely to the ArmLock sleeve intervention. A placebo, sham device, or standard treatment group would help distinguish the intervention’s effects from natural recovery or placebo effects. Third, self-reported compliance may be affected by recall bias or reporting inaccuracies, as participants tracked device usage in a diary. Future studies could incorporate wear-time sensors for more reliable adherence data. Fourth, lifestyle factors such as smoking and alcohol consumption were not measured in our study, highlighting a limitation. Future research on this device should consider assessing these variables to better understand their potential impact on outcomes. Fifth, although LE typically presents with an equal sex distribution [2], the number of male participants in our sample was twice that of female participants. Future research should consider refining recruitment strategies and materials to better engage and include female participants. Finally, the lack of long-term follow-up means it is unclear whether the observed benefits were sustained beyond 12 weeks. Building on the effect estimates generated by this study, future research with larger sample sizes should incorporate extended follow-up assessments at 3 to 6 months postintervention to evaluate the durability of intervention effects.

### Conclusion

This within-subjects repeated-measures study provides preliminary evidence supporting the ArmLock sleeve as a promising nonsurgical treatment for LE. Wearing the device daily for 30 minutes across 12 weeks led to statistically significant improvements in more than 80% of the outcome variables. Specifically, the intervention resulted in increased composite extensibility of the wrist and finger extensor muscles, enhanced PFG, and reduced pain intensity when lifting a 2-lb weight. Additionally, participants reported lower self-reported pain and functional disability. These findings suggest that the sustained muscle stretching provided by the ArmLock sleeve contributes to symptom reduction and improved functional performance in individuals with LE. However, future studies with a control group and a larger sample size are needed to provide more conclusive evidence regarding the device’s effectiveness.

## Abbreviations

ECRB: extensor carpi radialis brevis
FASTER: Framework for Accelerated and Systematic Technology-based Intervention Development and Evaluation Research
LE: lateral epicondylitis
NRS: Numerical Rating Scale
PFG: pain-free grip strength
PPT: pressure pain threshold
PRP: platelet-rich plasma
PRTEE: Patient-Rated Tennis Elbow Evaluation
RCT: randomized controlled trial
ROM: range of motion
